# Efficacy of PD-1/PD-L1 inhibitors against pretreated advanced cancer: a systematic review and meta-analysis

**DOI:** 10.18632/oncotarget.24163

**Published:** 2018-01-11

**Authors:** Hao Hu, Qian Zhu, Xian Shi Luo, Xiong Wen Yang, Hai Dong Wang, Chang Ying Guo

**Affiliations:** ^1^ Department of Thoracic Surgery, Medical College of Nanchang University, Nanchang, China; ^2^ Department of Biotherapy, Sun Yat-Sen University Cancer Center, Guangzhou, China; ^3^ Department of General Surgery, Medical College of Nanchang University, Nanchang, China; ^4^ Department of Lung Cancer Center, First People’s Hospital Chenzhou, Chenzhou, China; ^5^ Department of Thoracic Surgery, Jiangxi Province Tumor Hospital, Nanchang, China

**Keywords:** programmed cell death 1, programmed cell death-ligand 1, overall survival, progression-free survival, meta-analysis

## Abstract

**Background:**

Programmed cell death 1 (PD-1) and programmed cell death-ligand 1(PD-L1) inhibitors have captured our attention as new therapeutic options for several tumor types. Nonetheless, the differences in efficacy between PD-1/PD-L1 inhibitors and conventional treatments (chemotherapy or targeted therapy) in pretreated advanced cancer patients remain unclear.

**Materials and Methods:**

A systematic literature search was conducted to identify phase III randomized controlled trials (RCTs)-based investigations of PD-1(nivolumab, pembrolizumab)/PD-L1 inhibitors (atezolizumab) against pretreated advanced cancer. We evaluated these trials for inclusion, assessed each study’s risk of bias and selected relevant data for analysis.

**Results:**

The eligibility criteria were met by 5,093 patients from 8 phase III RCTs. PD-1/PD-L1 inhibitors significantly extended overall survival relative to the conventional treatment, expressed as hazard ratio [HR] (0.72, 95% CI, 0.66 to 0.77, *P* < 0.001) and median month difference (2.83 months, 95% CI, 1.87 to 3.78, *P* < 0.001). The progression-free survival HRs favored PD-1/PD-L1 inhibitors over conventional treatment (0.88; 95% CI, 0.82 to 0.95, *P* = 0.002), whereas median month difference was just the opposite (−0.69 months, 95% CI, −1.14 to −0.24, *P* < 0.001).

**Conclusions:**

Among selected patients with pretreated advanced cancer, PD-1/PD-L1 inhibitors, compared with conventional treatments (chemotherapy or targeted therapy), were associated with improvement in overall survival (2.83 months) but not progression-free survival. These findings will be important in considering PD-1/PD-L1 inhibitors in the treatment of pretreated advanced cancer and have implications for future study design.

## INTRODUCTION

Cancer, especially advanced cancer, is still a pressing worldwide health issue [1]. Although surgery, chemotherapy and radiation therapy have significantly improved overall clinical outcomes for localized cancer patients, advanced cancer patients present therapeutic challenges [2]. Over the past decades, targeted therapies, such as epidermal growth factor receptor (EGFR) and anaplastic lymphoma receptor tyrosine kinase (ALK) inhibitors [3, 4] in non-small-cell lung cancer (NSCLC), vascular endothelial growth factor (VEGF) pathway and mammalian target of rapamycin (mTOR) inhibitors in renal-cell carcinoma [5], V-raf murine sarcoma viral oncogene homolog B1 (BRAF) and mitogen-activated protein kinase kinase (MEK) inhibitors in melanoma [6] have changed the therapeutic landscape for these diseases. However, patients with advanced cancer whose disease progresses during or after first-line therapy have limited options with poor outcomes. Thus, novel treatment strategies to improve survival are warranted.

Immune checkpoint inhibitors, especially programmed cell death 1(PD-1) and programmed cell death-ligand 1(PD-L1) have captured our attention as new therapeutic options for patients with selected advanced cancer for which no effective treatment yet existed [7]. PD-1 receptor is expressed by activated T cells and is bound by PD-L1 and PD-L2, which are tumor-expressed ligands, to down-regulate T-cell activation and promote tumor immune escape [8]. PD-1/PD-L1 inhibitors disrupt PD-1/PD-L1-mediated signaling to reverse T-cell suppression and enhance endogenous antitumor immunity to unleash long-term antitumor responses in advanced cancer [9, 10]. Based on the initial trial findings, PD-1/PD-L1 inhibitors have shown clinical efficacies against many different solid and hematologic malignancies, including NSCLC, melanoma, renal cell cancer and others [9–12]. The documented promising outcomes with the PD-1/PD-L1 inhibitors have dramatically shifted our understanding of the overall approach to cancer therapy and represented a major step forward in cancer therapy. Currently, the following three PD-1/PD-L1 inhibitors have been approved by the United States (US) Food and Drug Administration (FDA): nivolumab is approved for renal cell cancers, melanomas, NSCLCs and classical Hodgkin lymphomas [13]; pembrolizumab is approved for melanomas, NSCLCs and head and neck cancers [14]; and atezolizumab is approved for urothelial carcinomas and NSCLCs [15].

Until now, several randomized controlled trials (RCTs), which compared the efficacies of PD-1/PD-L1 inhibitors to conventional treatments (chemotherapy or targeted therapy) against various pretreated advanced cancer, have been conducted [16–23]. However, the differences in efficacy between PD-1/PD-L1 inhibitors and conventional treatments (chemotherapy or targeted therapy) in pretreated patients with advanced cancer are inconclusive. Therefore, a pooled analysis of currently available studies may provide important and clinically useful information with respect to PD-1/PD-L1 inhibitors in the treatment of pretreated advanced cancer. Moreover, modern treatment-strategies for advanced cancer should focus on the rational delivery of systemic therapy and on the optimal combination strategies or sequence with novel agents such as cytotoxic T-lymphocyte-associated protein 4 (CTLA-4) inhibitors and other immunotherapy [24–26]. It will be of first importance in the design of informed clinical trials to determine which are the best outcomes achievable with systemic therapy and, ideally, for which patients. Quantifying the survival differences between the 2 treatment groups will be a compelling argument for further research. We therefore performed a systematic review and meta-analysis of phase III RCTs comparing PD-1(nivolumab, pembrolizumab)/PD-L1 (atezolizumab) inhibitors with conventional treatments in previously treated patients with advanced cancer.

## MATERIALS AND METHODS

### Search strategy

An independent review of the PubMed, Web of SCI, COCHRANE and ClinicalTrials.gov databases was performed from their inceptions to February 20, 2017. The search was conducted using the following keywords: “nivolumab,” “pembrolizumab” or “atezolizumab.” We also searched the American Society of Clinical Oncology conference proceedings, the European Society for Medical Oncology conference proceedings and Google Scholar to ensure that no eligible studies were overlooked. After the titles and abstracts were screened by two independent reviewers (H. H and Q. Z), the full texts from potentially relevant studies were retrieved to confirm the eligibility criteria. We also reviewed reference lists of original articles, review articles, and relevant books. When a duplicate publication of the same trial was found, the study with the most complete, recent, and updated report was included. All procedures were performed per the preferred reporting items for systematic reviews and meta-analyses statement [27].

### Selection and exclusion criteria

Studies that met the following criteria were included in the analysis: (1) prospective randomized III trials involving adult patients with pretreated advanced cancer, (2) random patient assignments to the study drug or non-PD-1/PD-L1 inhibitors control (chemotherapy or targeted therapy), (3) reports of the overall survival (OS) and/or progression-free survival (PFS) using a hazard ratio (HR) and differences in time (months). Reviews, editorials, case reports, phase I, phase II and non-randomized studies were excluded. Articles were excluded when they involved pediatric patients and patients with hematological malignancies. All articles on combination PD-1/PD-L1 inhibitors with other therapies in both the intervention and/or control cohorts were excluded.

### Data extraction and outcomes

The trial data were independently extracted by two reviewers (C Y. G and X S. L), and the results were compared to avoid bias from the data extraction process. The following information was obtained from each trial’s source: first author, tumor histology, number of patients for randomization, smoking status, PD-L1-positive status, median follow-up and treatment characteristics. We made efforts to contact the authors of these studies when the data were indeterminable.

The primary outcomes were OS and PFS. OS was defined as the time from randomization until death resulting from any cause. PFS was defined as the time from randomization to the time from randomization to first documented Response Evaluation Criteria in Solid Tumors (RECIST)-defined tumor progression or death from any cause. The summary measurements of OS and PFS were the HRs and/or median months of survival or time to disease progression or both, which were extracted from each study or obtained via contacting the authors. When data were unavailable and/or ambiguous, we attempted to contact the author of the study for clarification. Overall response rate (ORR) was not assessed because of the poor correlations between traditional RECIST ORR and the efficacy of immunotherapy agents [28].

The Cochrane Collaboration risk of bias assessment tool was conducted to evaluate the risk of bias [29]. Two reviewers (X W. Y and H D. W) independently extracted relevant data, which were verified by a third reviewer (C Y. G). Discrepancies among reviewers were resolved by consensus.

### Statistical analysis

A general variance-based method was used to estimate the summary HR and their 95% CIs, which were calculated to assess the benefit with respect to OS and PFS. When median survival times or median months of PFS were available, median differences were generated and combined. Random effects models were used to compute all the outcome measures investigated [30, 31] and heterogeneity has been taken into account across studies. Statistical heterogeneity in the results between studies included in the meta-analysis was quantified using the I^2^ statistic [100×(Q－df)/Q], which estimated the percentage of total variation across studies due to heterogeneity rather than chance, with I^2^ >50% indicating significant heterogeneity. The sources for heterogeneity were explored by conducting predefined subgroup analyses and meta-regression (in cases where the percentage of never smokers was given): treatment type (PD-1 inhibitors vs PD-L1 inhibitors), tumor type (NSCLC vs others), and PD-L1-positive status (unselected vs selected). A two-tailed *P* value of less than 0.05 was considered statistically significant. Funnel plots were performed to assess the potential publication bias. The Duval and Tweedie trim-and-fill test and the classic fail-safe N test were applied to define the extent of publication bias [32]. When publication bias is suspected, the Begg test [33] and Egger test [34] were used to quantify the level of bias. The Comprehensive Meta-analysis program (Version 2, Biostat, Englewood, NJ, USA) was used to performed statistical analyses.

## RESULTS

### Trial flow and study characteristics

**Figure 1:**
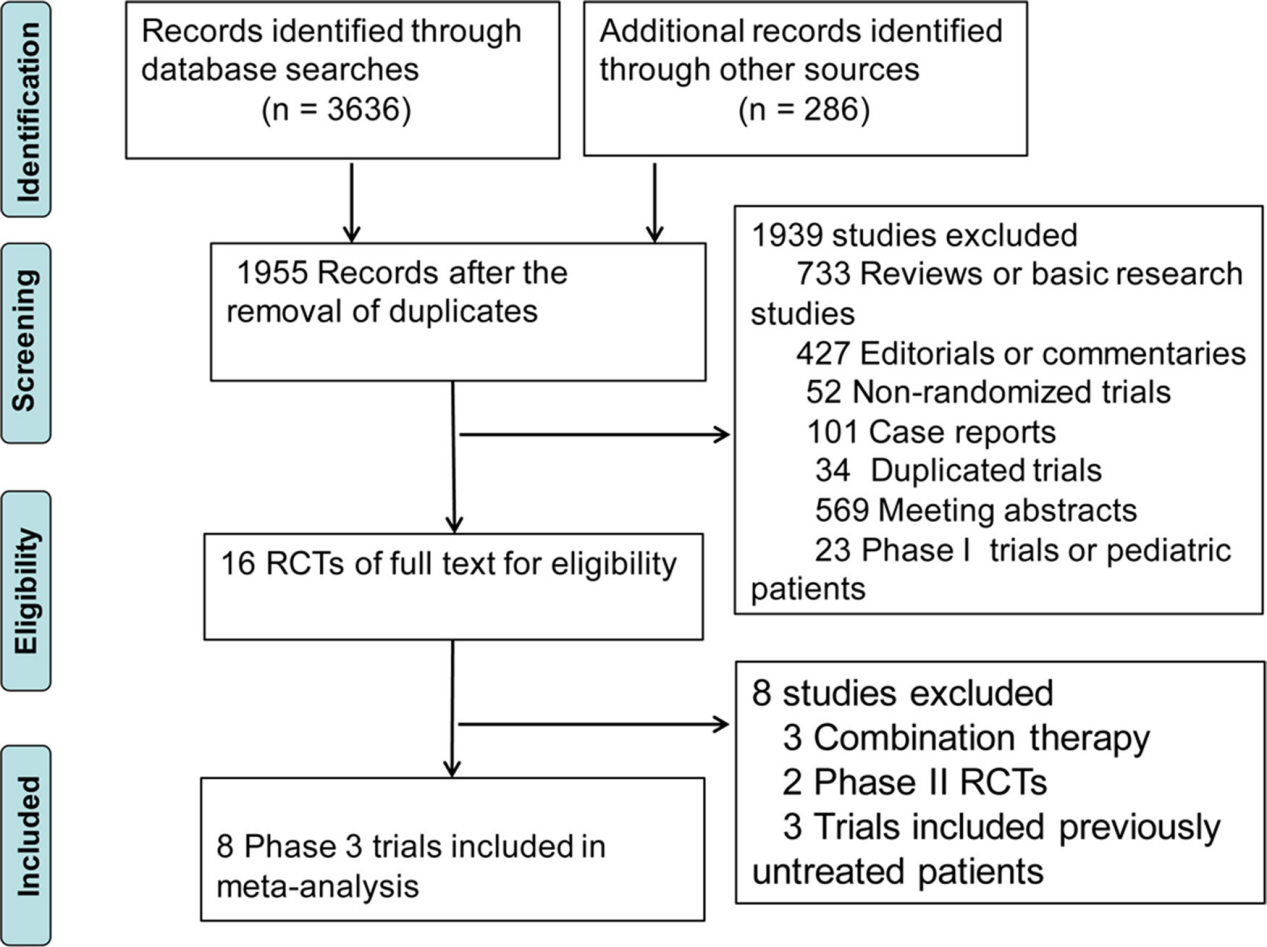
Selection of randomized controlled trials (RCTs) included in the meta-analysis

As shown in Figure 1, the full texts of 16 trials were retrieved for the detailed evaluation. After further review, 3 trials were excluded by their combinatorial use of PD-1/PD-L1 inhibitors with other therapies [35–37]. Moreover, 2 phase II RCTs [38, 39] and 3 trials including previously untreated patients [40–42] were excluded. Ultimately, 8 trials that involved 5,093 advanced cancer adult patients were analyzed [16–23].

7 trials used PD-1 inhibitors including 5 trials with nivolumab [16–20], 2 with pembrolizumab [21, 23]; one trial used PD-L1 inhibitors (atezolizumab) [22]. Four RCTs were conducted for NSCLCs [16, 17, 21, 22]; 4 trials were managed for others (including melanoma [19], renal-cell carcinoma [18], head and neck cancers [20] and urothelial carcinoma [23]). One study [21] of NSCLCs reported 2 treatment arms with different regimens (pembrolizumab 2 mg/kg vs 10 mg/kg). These cohorts were recorded separately, resulting in a total of 9 independent study cohorts (3 pembrolizumab, 5 nivolumab, and 1 atezolizumab cohorts) from 8 eligible studies for a meta-analysis. The proportion of never smoker varied extensively between studies, ranging from 7% to 39% of total cancers. Among all the included clinical trials, only one trial recruiting selected PD-L1-positive (PD-L1 expression on at least 1% of tumor cells) patients [21].

All trials recruited patients with a performance status of 0 through 2 in the Eastern Cooperative Oncology Group (ECOG), apart from 1 trial [18], which included patients with a Karnofsky performance status (KPS) of at least 70. Meanwhile, all trials included mostly white patients (rang from 86% to 99%). Tumor response in all studies was assessed according to RECIST version 1.1. The PD-1/PD-L1 inhibitors treatment arm was administered as monotherapy.

All trials used commonly recommended second- or later-line therapeutic regimen as the control group (docetaxel in NSCLC: category 2A [43]; everolimus in clear-cell renal-cell carcinoma: category 1 [44]; dacarbazine or paclitaxel combined carboplatin in melanoma: category 2A [45] in the National Comprehensive Cancer Network guideline), except one trial [23], which used paclitaxel, docetaxel or vinflunine as a control drug because of there was no internationally accepted therapeutic regimen after standard first-line treatment. The characteristics of these trials are presented in Table 1.

**Table 1:** List of study characteristics

First Author	Histology	No. of Total Patients (% Female)	Never Smoker (%)	PD-L1 Status	Median Follow-up (months)	Treatment Type	Intervention Treatment	Control Treatment
Borghaei,^16^	NSCLC	582(48)	20	unselected	20.1	PD-1 inhibitors	nivolumab 3mg/kg q2w	docetaxel
Brahmer,^17^	NSCLC	272(18)	7	unselected	17.5	PD-1 inhibitors	nivolumab 3mg/kg q2w	docetaxel
Motzer,^18^	RCC	821(23)	NA	unselected	23.5	PD-1 inhibitors	nivolumab 3mg/kg q2w	everolimus
Weber,^19^	Melanoma	631(35)	NA	unselected	8.4	PD-1 inhibitors	nivolumab 3mg/kg q2w	(dacarbazine, paclitaxel) + carboplatin
Ferris,^20^	HN	361(18)	16	unselected	5.1	PD-1 inhibitors	nivolumab 3mg/kg q2w	methotrexate, docetaxel, or cetuximab
Herbst,^21,a^	NSCLC	1034(38)	18	selected PD-L1 +	13.1	PD-1 inhibitors	pembrolizumab 2mg/kg q3w	docetaxel
	NSCLC	(38)	17	selected PD-L1+	13.1	PD-1 inhibitors	pembrolizumab 10mg/kg q3w	docetaxel
Rittmeyer,^22^	NSCLC	850(39)	20	unselected	21	PD-L1 inhibitors	atezolizumab 1200 mg q3w	docetaxel
Bellmunt,^23^	UC	542(26)	39	unselected	14.1	PD-1 inhibitors	pembrolizumab 200 mg q3w	paclitaxel, docetaxel or vinflunine

NSCLC: non-small-cell lung cancer; RCC: Renal-Cell Carcinoma; HN: Squamous-Cell Carcinoma of the Head and Neck; UC: Urothelial Carcinoma. a: The studies provided 2 independent cohorts treated with different regimens (pembrolizumab 2mg/kg q3w vs 10mg/kg q3w).

### PD-1/PD-L1 inhibitors significantly prolongs OS

**Figure 2:**
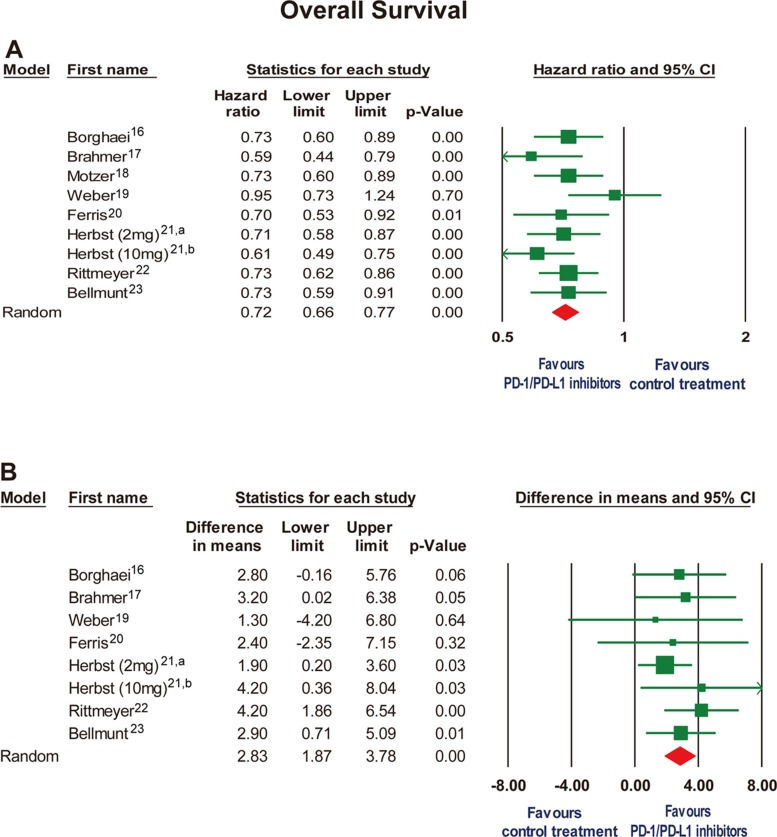
Forest plot for overall survival expressed as (**A**) hazard ratio and (**B**) mean differences in months when median overall survival data were reported. CI, confidence interval. a: the study of pembrolizumab 2 mg/kg every 3 weeks by Herbst et al^21^. b: the study of pembrolizumab 10 mg/kg every 3 weeks by Herbst et al^21^.

We analyzed HRs and the differences in medians for OS separately. OS was significantly longer with PD-1/PD-L1 inhibitors than with conventional treatments, both expressed as HR (0.72, 95% CI, 0.66 to 0.77, *P* < 0.001) and median month difference (2.83 months, 95% CI, 1.87 to 3.78, *P* < 0.001, Figure 2). No or minimal heterogeneity was detected in the analysis of both HR and median month difference in OS (I^2^, 5%; I^2^, 0%; respectively). Moreover, there was no publication bias for OS expressed as HR and median month difference (Supplementary Figure 1).

### Association of PD-1/PD-L1 inhibitors with PFS

**Figure 3:**
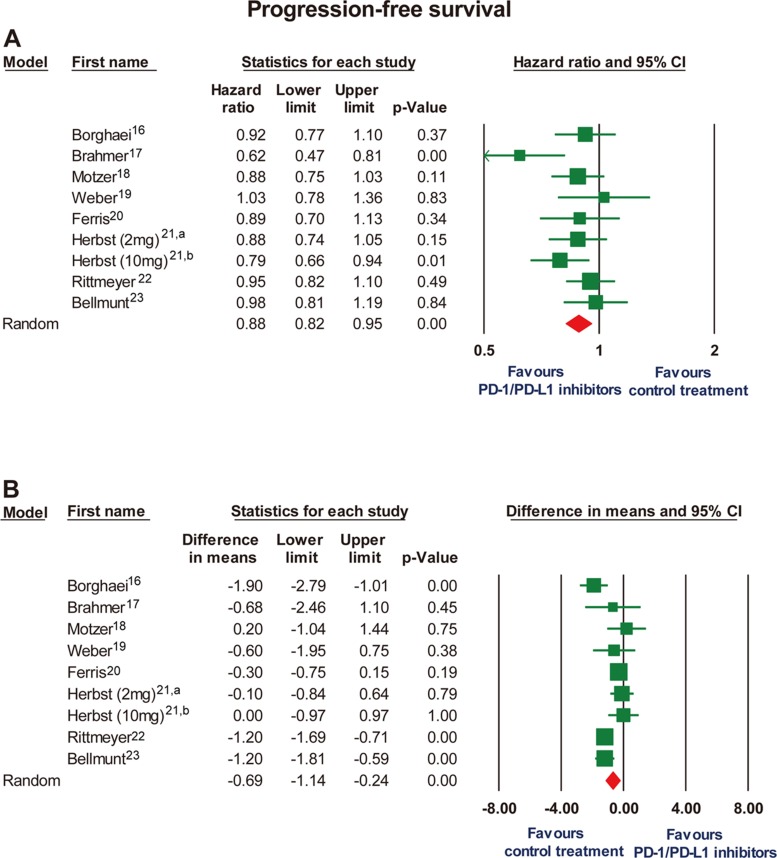
Forest plot for progression-free survival expressed as (**A**) hazard ratio and (**B**) mean differences in months when median progression-free survival data were reported. CI, confidence interval. a: the study of pembrolizumab 2 mg/kg every 3 weeks by Herbst et al^21^. b: the study of pembrolizumab 10 mg/kg every 3 weeks by Herbst et al^21^.

We further analyzed HRs and the differences in medians for PFS. Significant prolongation of PFS was observed in PD-1/PD-L1 inhibitors group expressed as HR (0.88; 95% CI, 0.82 to 0.95, *P* = .002), whereas PD-1/PD-L1 inhibitors suggested shorter median PFS expressed in months (difference, −0.69 months, 95% CI, −1.14 to −0.24, *P* < .001) (Figure 3). No significant heterogeneity was observed in evaluating HR for PFS (I^2^ = 30.2%). However, a significant statistical heterogeneity was noted in the analysis of median month difference in PFS (I^2^ = 65.3%). No or minimal publication bias for the outcome PFS expressed as HR and median month difference was noted (Supplementary Figure 2). The Duval and Tweedie trim-and-fill test with adjusted values suggested the observed point estimate was not significant altered (data not shown).

### Subgroup analyses

Subgroup analyses were carried out to explore the heterogeneity in the terms of median month difference for PFS because the significant heterogeneity existed within included studies (Table 2). There were no significant differences between the subgroups by treatment type and tumor type (*P* values for subgroup difference: type of treatment, 0.35; and type of tumor, 0.58, respectively). However, the subgroup result of PD-L1-positive status appeared to be discordant: trials recruiting selected PD-L1-positive patients showed no significant difference in median month difference of PFS between PD-1/PD-L1 inhibitors with control group (difference, - 0.06; 95% CI, −0.94 to 0.83). On the other hand, in trials recruiting patients with unselected PD-L1 expression, PD-1/PD-L1 inhibitors demonstrated a shorter median PFS (difference, −0.88; 95% CI, −1.36 to −0.41) compared with control treatment. PD-L1-positive status can partly explain the heterogeneity between the trials, but the subgroup difference did not yet reach the level of statistical significance (*P* = 0.11). In a meta-regression analysis regarding smoking status, there was no correlation between the percentages of never smokers and the standardized mean difference for PFS (*P* = 0.29); Supplementary Figure 3.

**Table 2:** Subgroup analyses for progression-free survival in median month difference

Type of treatment	No. of Trials	Progression-Free Survival (median month difference, 95% CI)
PD-1 inhibitors	8	−0.60 (−1.09 to −0.10)
PD-L1 inhibitors	1	−1.20 (−2.36 to −0.04)
Subgroup difference: *P* = 0.35		
Type of tumor		
NSCLC	5	−0.81 (−1.46 to −0.16)
others	4	−0.54 (−1.25 to 0.18)
Subgroup difference: *P* = 0.58		
PD-L1 expression status		
unselected	7	−0.88 (−1.36 to −0.41)
selected PD-L1-positive	2	−0.06 (−0.94 to 0.83)
Subgroup difference: *P* = 0.11		

Abbreviations: PD-1, programmed cell death 1; PD-L1: programmed cell death ligand 1; NSCLC, non–small cell lung cancer.

### Bias assessment

**Figure 4:**
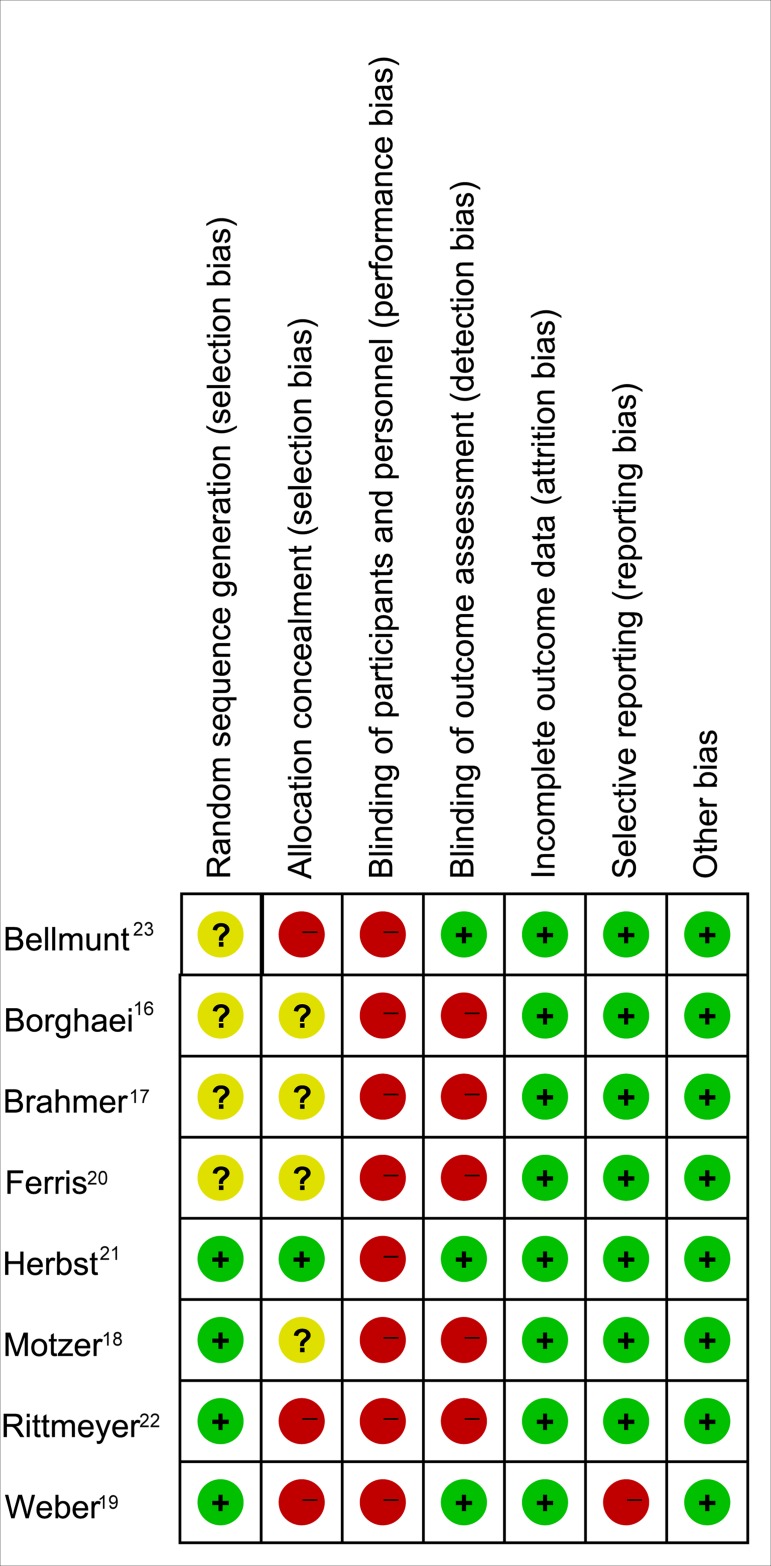
The assessment of each study’s risk of bias using the cochrane collaboration risk of bias assessment tool

The result of risk of bias assessment is provided in Figure 4. Selection bias could be detected due to inadequate concealment of allocations in three trials [19, 22, 23], however, selection bias was low in others trials, or were not reported. With the exception of three studies with a low risk of bias [19, 21, 23], performance bias and detection bias could be noted in all studies, which, in most cases, was because all trials were open-labeled. Attrition bias, reporting bias, and other bias were generally low in all studies, but reporting bias could be observed in one trial [19].

## DISCUSSION

We performed the meta-analysis with a focus on investigating the efficacy difference between PD-1/PD-L1 inhibitors and conventional therapies in pretreated advanced cancer patients. This study included all published 8 well-conducted, good-quality, phase 3 RCTs incorporating 5,093 patients with pretreated advanced cancer. Considering PD-1/PD-L1 inhibitors blockade PD-1/PD-L1 pathways by activating an immune response directed against cancer, it exhibit antitumor activity in the form of a cytostatic, rather than a cytotoxic effect [28]. Thus, PD-1/PD-L1 inhibitors could slow or stop tumor development, growth and metastasis without shrinking existing tumors. Moreover, PD-1/PD-L1 inhibitors have delayed effect and atypical kinetics and thus late responders, or who experience a response after initial tumor growth may be classified erroneously as non-responders [28]. Consequently, the traditional ORR was not analyzed in present study. Our study demonstrated that among selected patients with advanced, previously treated cancer, PD-1/PD-L1 inhibitors, compared with conventional treatments (chemotherapy or targeted therapy), were associated with improvement in OS but not PFS.

Present pooled analysis suggested that PD-1/PD-L1 inhibitors prolong OS (2.83 months) compared with conventional treatments, however, the corresponding reduction of median PFS with PD-1/PD-L1 inhibitors were 0.69 months. The apparent discordance between PFS and OS have been commonly noted [16, 20, 22, 23], which can be attributed to the pseudoprogression, delayed antitumor activity, or antitumor immune activation beyond progression that might be sustained by continued treatment [28]. The PFS HRs favored PD-1/PD-L1 inhibitors over conventional treatments, whereas median month difference is just the opposite. The apparent discrepancy between the HR and median month differences in PFS maybe attribute to the delay antitumor activity with PD-1/PD-L1 inhibitors that may be typical with the drug classes, as indicated by the authors [16, 18, 20, 23]. The better toxicity profile of PD-1/PD-L1 inhibitors compared with conventional treatments has been demonstrated in several meta-analyses [46, 47], which may directly correlates with its targeted mechanism of action. Therefore, in selected advanced cancer patients whose disease progresses after standard first-line treatment, PD-1/PD-L1 inhibitors could be a preferable therapeutic schedule over conventional therapies. However, it is not yet clear whether the finding could be applied to other tumors and first-line setting and thus the unanswered questions suggest areas that are in need of further study.

PD-1 inhibitors block the PD-1 interaction with both PD-L1 and PD-L2. However, PD-L1 inhibitors block interactions between PD-L1 and both PD-1 and CD80 whereas the latter interaction may down-modulate T-cell responses [9]. Moreover, PD-L1 inhibitors didn’t block interactions between PD-L2 and PD-1, which would still preserve the function of PD-L2 while relieving PD-1 mediated suppression. Therefore, in theory, PD-1 inhibitors should have better antitumor efficacy than PD-L1 inhibitors [48]. However, this theoretical advantage of PD-1 inhibitors over PD-L1 inhibitors has not yet translated into a clear clinical difference. We found that PD-1 inhibitors didn’t produce a statistically significant improved effect compared with PD-L1 inhibitors (Table 2), but this can be attributed to the limited amount of studies available for subgroup analysis. A randomized head-to-head comparison testing whether PD-1 inhibitors are superior to PD-L1 inhibitors is needed.

Efforts to find the appropriate biomarker to identify which patients would benefit most from PD-1/PD-L1 inhibitors is a crucial personalized-medicine approach to increase response rates and many different predictive biomarkers are evaluated. Of these, the relationship between PD-L1 expression on tumor cells and outcome to PD-1/PD-L1 inhibitors has been extensively investigated but are still inconclusive, which could partly be due to the function of complex interactions between tumors and the immune system [10, 42, 49]. In present study, the trials screening patients with PD-L1-positive were associated with longer trend of PFS with PD-1/PD-L1 inhibitors. Indeed, the presence of PD-L1 expression may indicate a trend of enhanced antitumor activity favoring PD-1/PD-L1 inhibitors, however, several factors such as the use of distinct assay, different screening thresholds and measure expression on different cells within the tumor microenvironment could bias the findings [50]. Moreover, some patients with lack of PD-L1 expression may still benefit from PD-1/PD-L1 inhibitors and thus it should not preclude use of these agents [17, 22]. Additionally, the proportion of actually PD-L1 positive tumor cells may quite low in many tumors [51]. More importantly, dynamic changes in PD-L1 expression were clear indicated where adaptive immune resistance is concerned [52]. Thus, these have limited the potential utility of PD-L1 expression as the only predictive marker to predict outcome to PD-1/PD-L1 inhibitors. At the same time, several studies focused on other potential biomarkers such as mismatch repair deficiency [53], mutational load [54] and the composition of the gut microbiome [55] and its interaction with PD-1/PD-L1 inhibitors need to be further explored. In addition to optimal dosing and duration of treatment, further investigation into optimal predictive/prognostic factor and ideal patient populations in order to specifically benefit those that are most likely to benefit is needed.

PD-1/PD-L1 inhibitors have shown promising activity against many tumor types, whereas only a subset of patients can respond to such mono-therapy [9, 10]. The exploration of the rational therapeutic strategies for combination with PD-1/PD-L1 inhibitors to produce a durable anti-tumor response in patients who do not respond to mono-therapy might be needed. Currently, there have numerous studies investigating others possible synergies of combinations with PD-1/PD-L1 inhibitors, including combinations with immune-modulatory targets, chemotherapy, radiation therapy, or targeted therapy. Encouraging results from combination treatment with PD-1 and CTLA-4 inhibitors have been observed in melanoma and NSCLC [35, 56]. Although such combination appears to increase toxicities, the most common toxicities are immune-related adverse effects, which can be severe but largely manageable with immunosuppressant [25]. Notwithstanding, the challenges associated with developing rational combination strategies need to address. On the one hand, intensified anti-tumor activity without a corresponding increase in serious toxicities by the appropriate timing, dosage and sequencing of regimens will probably be critical to the success of combinatorial approaches. On the other hand, there have the myriad possibilities for combination therapies and thus a simple, robust approach of stratification and identification is warranted. For example, it has been suggested that expression of tumor PD-L1, tumor infiltrating lymphocytes infiltration and certain genetic signatures of tumor cells could support stratify patients and identify about the optimal combination strategy in the treatment of each tumor type [52].

Two meta-analyses have tried to explore the efficacy of PD-1/PD-L1 inhibitors. One meta-analysis included only 3 trials of PD-1/PD-L1 inhibitors [57] and the other included only 4 trials [58]. Furthermore, these studies included 1 small phase II trials among them, and only NSCLCs patients were included. Additionally, quantitative the survival differences between PD-1/PD-L1 inhibitors and conventional therapies were not conducted and information from new trials hasn’t been incorporated. Our current meta-analyses including 5,093 patients from high quality phase 3 RCTs is thus the largest meta-analysis of PD-1/PD-L1 inhibitors in pretreated advanced cancer patients and provides a more reliable and higher quality evidence for judging the efficacy of treatment with PD-1/PD-L1 inhibitors. Most importantly, our study is the first meta-analysis to report on quantitative the survival differences with any immune checkpoint inhibitors, as well as PD-1/PD-L1 inhibitors.

Inclusion of well-conducted, good quality, phase 3 RCTs are the strengths of our study. The lack of heterogeneity in the assessment of OS represents a well selection of studies. Furthermore, subgroup analyses were all pre-planned and limited to a minimum. Inclusion of large number of patients and recent studies are other strengths. However, our study encountered several limitations relevant to this meta-analysis. First, the analysis was based on extracted data and not on individual patient data. Therefore, the interpretations of the results needed to be carefully performed, especially for the specific relationships in the subgroup analysis. Further studies using the individual patient data will be necessary to assess outcomes, such as the primary outcomes and subgroup interactions. Second, this meta-analysis involved the different treatment schedules between the trials. In two trials, patients received pembrolizumab per the following schedules: 2 mg/kg, 10 mg/kg [21] or 200 mg [23] every 3 weeks. These different treatment schedules contributed to the increased clinical heterogeneity in this meta-analysis. However, the clinical heterogeneity may improve the generalizability of our observations. What’s more, given the paucity of data and power limitations, we could not focus on one specific tumor. Finally, there are a relative limited number of studies available for analysis and more studies are still needed.

In conclusion, our meta-analysis identified that in selected pretreated advanced cancer patients, OS was significantly longer (2.83 months) with PD-1/PD-L1 inhibitors than with conventional treatments, whereas median PFS was just the opposite (−0.69 months). These findings provide important and clinically useful information with respect to PD-1/PD-L1 inhibitors as novel treatment strategies to improve patient overall survival, as well as could be useful for the design of future studies.

## SUPPLEMENTARY MATERIALS FIGURES


