# Supplementary material for: Efficacy of PD-1/PD-L1 inhibitors against pretreated advanced cancer: a systematic review and meta-analysis
Source: Oncotarget. 2018 Jan 11;9(14):11846–57. doi: 10.18632/oncotarget.24163 (PMC5837741; doi:10.18632/oncotarget.24163)
Supplement: Supplementary file 1 [file oncotarget-09-11846-s001.pdf]

Efficacy of PD-1/PD-L1 inhibitors against pretreated advanced cancer: A systematic review and meta-analysis

SUPPLEMENTARY MATERIALS

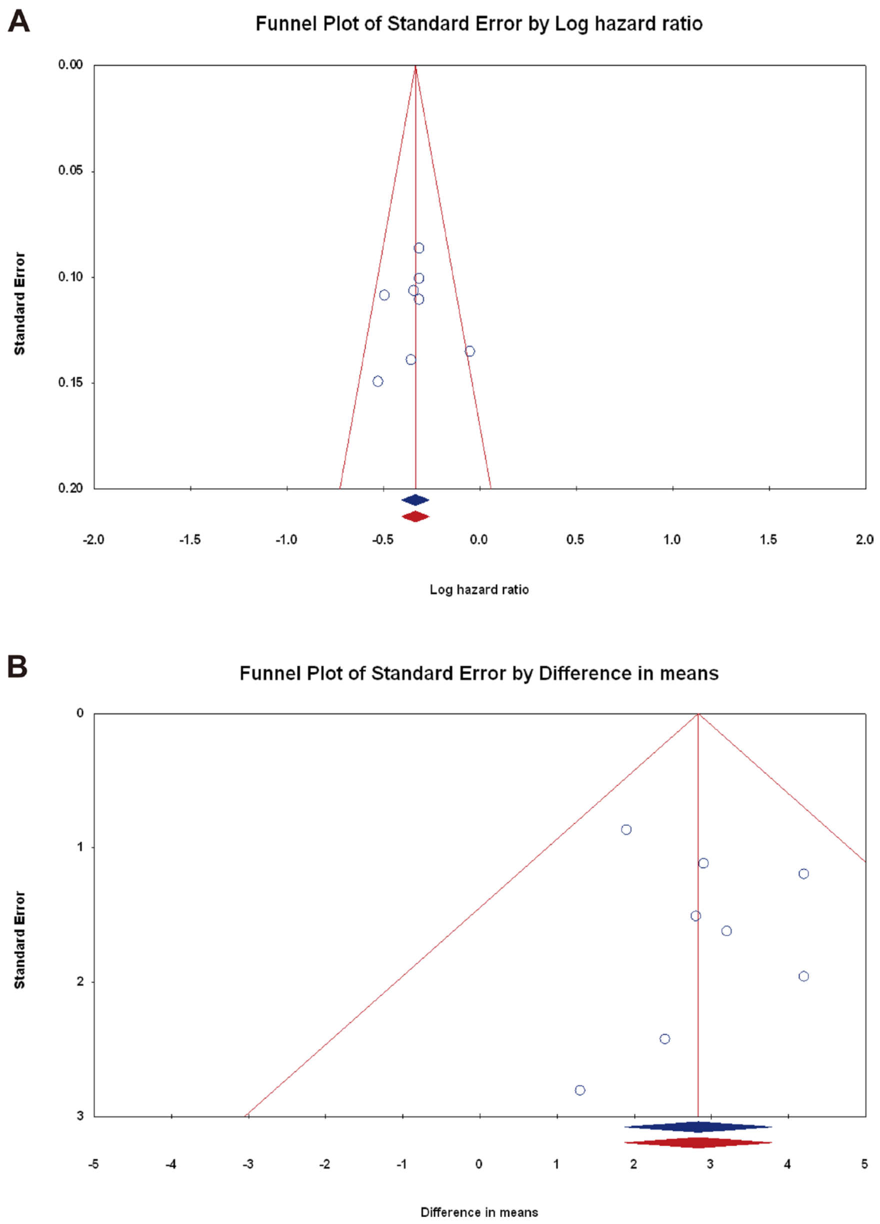

Supplementary Figure 1: Duval and Ttweedie trim-and-fill analysis. Funnel plots depicting potential presence of publication bias when analyzing overall survival as (**A**) hazard ratios and as (**B**) median month difference.

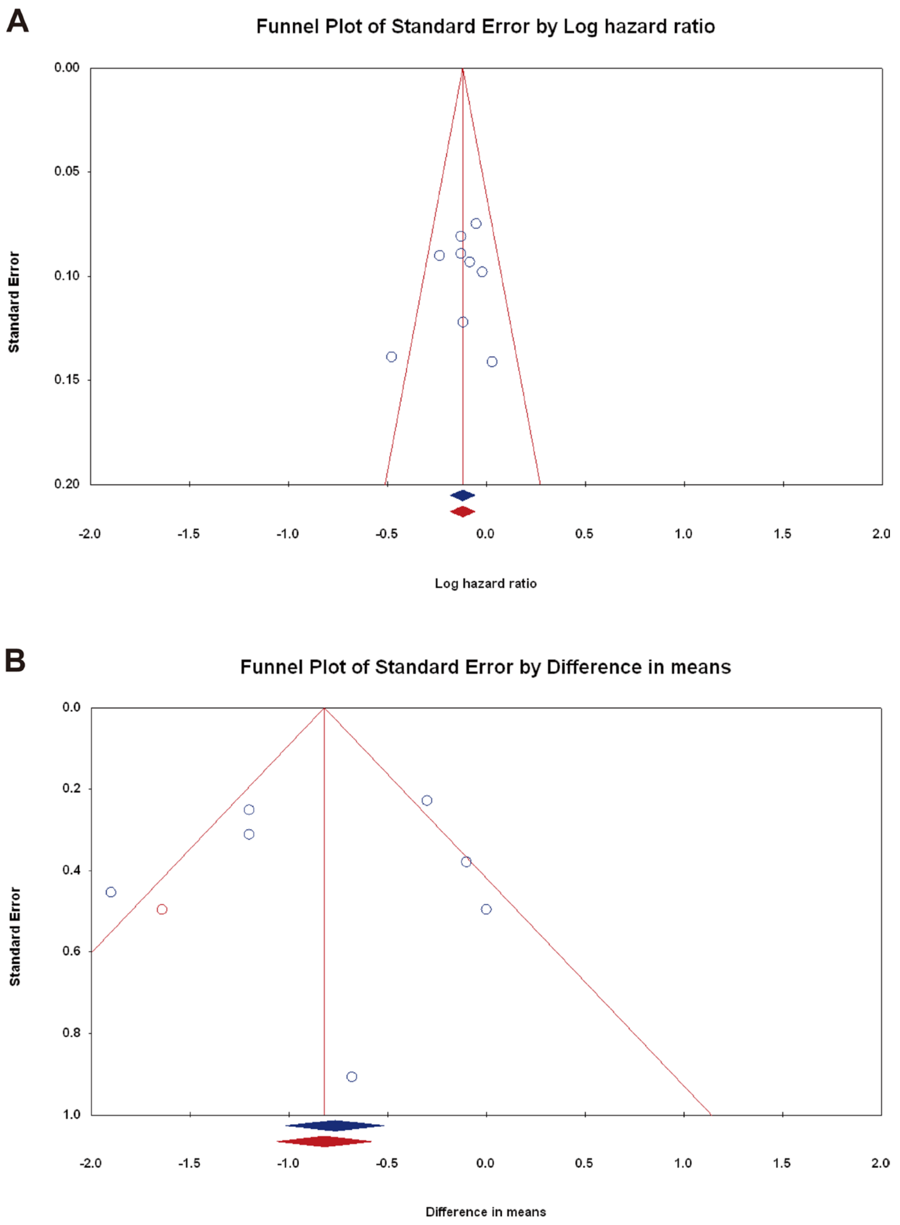

Supplementary Figure 2: Duval and tweedie trim-and-fill analysis. Funnel plots depicting potential presence of publication bias when analyzing progression-free survival as (**A**) hazard ratios and as (**B**) median month difference.

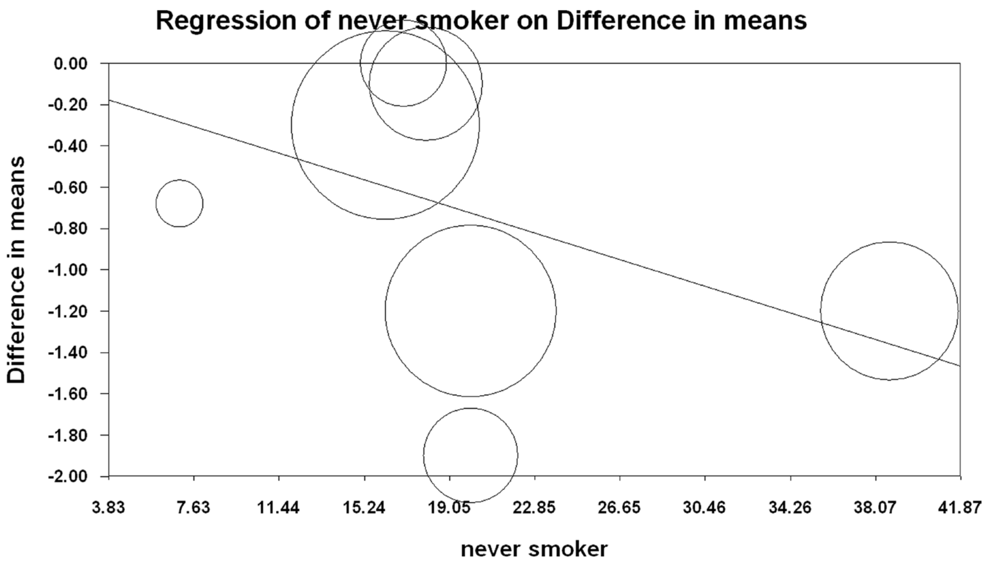

Supplementary Figure 3: Meta-regression analysis suggesting the correlation between smoking status (percentage of never smokers) and difference in median progression-free survival in months.
